# High performance of a unique mesoporous polystyrene-based adsorbent for blood purification

**DOI:** 10.1093/rb/rbw038

**Published:** 2016-12-24

**Authors:** Jian Chen, Wenyan Han, Jie Chen, Wenhui Zong, Weichao Wang, Yue Wang, Guanghui Cheng, Chunran Li, Lailiang Ou, Yaoting Yu

**Affiliations:** Key Laboratory of Bioactive Materials, Ministry of Education, College of Life Sciences, Nankai University, Tianjin 300071, China

**Keywords:** polystyrene adsorbent, mesoporous, toxins removal, blood purification

## Abstract

A multi-functional polystyrene based adsorbent (NKU-9) with a unique mesoporous and a high surface area was prepared by suspension polymerization for removal of therapeutic toxins in blood purification. The adsorbent produced had an almost equal amount of mesopore distribution in the range from 2 to 50 nm. The adsorption of serum toxins with different molecular weights were examined by in vitro adsorption assays and compared with some clinical currently used adsorbents such as HA-330, Cytosorb and BL-300 which are produced by China, America and Japan, respectively. Test results indicated that the adsorption rate for pentobarbital by NKU-9 was 81.24% which is nearly as high as HA-330 (81.44%). The latter adsorbent is currently used for acute detoxification treatment in China. To reach adsorption equilibrium, NKU-9 was faster than HA-330, which implies short treatment time. For the removal of middle molecular toxins such as β2-microglobulin (98.88%), NKU-9 performed better adsorptive selectivity than Cytosorb (92.80%). In addition, NKU-9 showed high performance for the removal of albumin-bound toxins (e.g., bilirubin), and its adsorption rate for total bilirubin (80.79%) in plasma was 8.4% higher than that of anion exchange resin BL-300 which is currently used to eliminate bilirubin in clinic. Therefore, our results indicate that the newly developed adsorbent with a wide distribution and almost equal amount of mesopores is a multifunctional adsorbent for high efficient removal of serum toxins with different molecular weights which might be an excellent blood purification adsorbent especially to treat diseases that conventional medical methods are low or not efficient.

## Introduction

Organ failure often is the end-stage result of various diseases and a range of potentially toxic substances accumulate in the blood stream of the patient, causing high morbidity and mortality [1, 2]. In the last decades, hemoperfusion based on adsorbents have been introduced for highly specific and efficient removal of therapeutic toxins which can cure or alleviate the diseases, thus permitting organ transplantation [3–5].

Human diseases, associated with toxins accumulation, are often complicated, and the therapeutic toxins are composed of a range of substances with different molecular weights [6, 7]. For example, metabolites associated with uremia include water-soluble compounds of low molecular weight (e.g. creatinine, urea), middle molecular toxins (e.g. parathyrin, β2-microglobulin) and albumin-bound toxins (e.g. bilirubin *p*-cresyl-sulfate). These are small molecules but predominantly non-covalently bound in plasma to albumin (66 kDa) [8, 9]. Multi-functional adsorbents are medically necessary, because they can simultaneously remove various serum toxins with different molecular weights by means of one-shot treatment. But in practice, currently available adsorbents always have a concentrated pore size distribution in a narrow range and remove specific toxins with molecular weight selectivity [10–14].

As for adsorption of low molecular weight toxins, activated charcoal and hyper-crosslinked adsorption resins could be a good choice, such as HA-330 (made in China) [12, 15]. These two kinds of adsorbents have a large number of micropores and high specific surface area, resulting in their excellent adsorption properties for low molecular weight toxins. However, they also have some significant drawbacks, such as inadequate removal of middle molecular weight substances (cytokines and β2-microglobulin) because of space conﬁnement imposed by small pore sizes, mostly less than 2 nm. A hemoperfusion cartridge used for cytokine removal by molecular sieve mechanism is the CytoSorb^TM^ cartridge (Cytosorbents, NJ, USA) and their hemoadsorption beads are made of polystyrene–divinylbenzene porous particles with a biocompatible polyvinyl–pyrrolidone coating [16]. The CytoSorb adsorbent is efficient in removing middle molecular substances, such as cytokines and β2-microglobulin [17–19]. However, there is a problem to reduce non-specific adsorption of plasma proteins, for example albumin. In terms of albumin-bound toxins removal, ion exchange resins are currently used in clinical practice, For example, bilirubin adsorption column Medisorba BL-300 (Kuraray Medical, Osaka, Japan) is applied for the treatment of hyperbilirubinemia [20]. The adsorbent binds bilirubin with high efficiency; however, there are indications that positively charged polymers also remove coagulation factors and thus lead to disturbances of the coagulation system. Another system is the molecular adsorbent recirculating system (MARS) which is a commercially available artificial liver support. It uses 20% human albumin continuously circulating through two columns, one of charcoal and the other of an anion exchanger to efficiently remove different molecular weight toxins, especially those bound to albumin [21–23]. However, it is an expensive treatment for the vast majority of patients.

The aim of the present study was to prepare a multi-functional adsorbent, having a wide pore distribution and an equal amount of mesopore size from 2 to 50 nm and a high surface area. It can remove different toxins with high efficiency including albumin-bound toxins. The adsorbent has a high performance, similar to MARS, but is very cost-effective. Experimental results indicate that our newly developed NKU-9 adsorbent having a unique mesoporous structure can efficiently remove therapeutic toxins with different molecular weights in hemoperfusion. Therefore, it has a high potential to be used clinically in blood purification especially to treat those diseases whose pathological mechanism is complicated or unknown.

## Materials and methods

### Chemicals and plasma

Bilirubin and cholic acid were purchased from Sigma-Aldrich (St. Louis, USA), bovine serum albumin (fraction V) and hen egg lysozyme were from Unite Stars (Tianjin, China). Human plasma from hyperbilirubinemia or dialysis patients was provided by the Second People's Hospital of Tianjin (Tianjin, China) and used with the patient’s permission. Pentobarbital (reagent grade) was obtained from Beijing Chemical Reagent Company (Beijing, China).

### Adsorbent preparation

Styrene divinylbenzene copolymer was prepared by suspension polymerization in the presence of an inert solvent (porogen) [24]. Pore size and particle size were controlled by a variation of the porogen and modulator. After polymerization, the adsorbent signified as NKU-9 was purified in a chromatography column by subsequent washing with water, acetone and ethyl alcohol. Then the adsorbent was thoroughly washed in distilled water to remove any remaining solvent, and stored in distilled water at 4°C for further use. Adsorbent, HA-330 (Jafron, Zhuhai, China), Cytosorb (Cytosorbents, NJ, USA) and BL-300 (Kuraray Medical, Osaka, Japan) were adopted as a comparison to NKU-9.

### Adsorbent characterization

The morphology and microstructure of the adsorbent was analyzed by scanning electron microscope (SEM, QUANTA 200, Czech). SEM images were obtained after drying the adsorbent beads in vacuum at 120–130°C for 12 h. The N_2_ adsorption/desorption isotherms were measured on Micromeritics ASAP 2460 at liquid nitrogen temperature (77 K). Specific surface areas were calculated by BET method using adsorption isotherms and pore size distributions were calculated by DFT method [25–27].

### Adsorption studies in static batch experiments

Bilirubin, cholic acid, pentobarbital and lysozyme solutions were prepared in phosphate buffer solution (PBS). Due to the poor solubility of bilirubin in water, it was initially dissolved in a small volume of DMSO and 0.1 M NaOH, and cholic acid powder was dissolved in 3% ethanol solution then added to PBS. In order to simulate the state of bilirubin in plasma, albumin (15 mg/ml) was added to the above PBS-bilirubin solution, and stirred for 60 min at room temperature to allow the binding of bilirubin to albumin. Since bilirubin is light sensitive, all bilirubin adsorption experiments were carried out in a dark room, and the solution was used immediately after preparation.

Prior to use for adsorption experiments, the adsorbent was swelled fully in 0.9% saline solution. Aliquots (wet volume) of adsorbent were incubated with appropriate volumes of the adsorbate solutions at 37°C with constant shaking. During the progress of adsorption, samples of bilirubin, bile acid, pentobarbital, lysozyme and serum albumin were drawn at pre-set time, and stored at 4°C until quantitative analysis was performed. All the adsorption experiments were conducted in triplicates.

### Quantitative analysis of the adsorbate solutions

Bilirubin analysis was conducted according to the literature [28]. Briefly, test sample was reacted with diazo reagents and the color solution formed was measured photometrically. The bile acid concentration was determined quantitatively by spectrophotometer at 387 nm after 1 ml of samples reacted with 6.5 ml of 45% sulfuric acid solution in a hot water bath (70°C) for 1 h [29]. The concentrations of pentobarbital (240 nm), albumin (280 nm) and lysozyme (280 nm) were also determined quantitatively by spectrophotometer.

### Adsorption assays in human plasma

For adsorption studies of middle molecular weight toxins (cystatin C and β2-microglobulin) in human plasma from dialysis patients, 0.5 ml (wet volume) of adsorbent were incubated with 4 ml of plasma under constant shaking for 2 h at 37°C. Then, the supernatants were collected and analysis was performed in Teda Hospital of Tianjin.

Human plasma was spiked with TNF-α and IL-6 at a concentration of 1000 pg/ml and used to test the adsorption capacity of NKU-9. Batch tests in triplicates were conducted by incubating 0.5 ml (wet volume) of adsorbent with 3.5 ml of spiked plasma. After 2 h of incubation at 37°C, samples were centrifuged. The supernatants were collected and stored at 4 °C and analysis was performed with ELISA kits (Quantikine R&D System).

For adsorption studies of bilirubin in human plasma, 0.5 ml (wet volume) of adsorbent were incubated with 2.5 ml of human plasma with high levels of bilirubin from liver failure patients for 2 h, at 37°C under constant shaking. After incubation, blood chemistry analyses were performed in Teda Hospital of Tianjin which included bilirubin, total bile acid, etc. All adsorption assays in human plasma were conducted in triplicates.

### Adsorption of different toxins in a mixed solution

For adsorption studies in a mixed solution, BSA solution was spiked with the following different molecular weight toxins: bilirubin (200 mg/l, *M*_W_ = 584.66 Da), cholic acid (200 mg/l, *M*_W_ = 408.58 Da) and IL-6 (1000 pg/ml, *M*_W_ = 21.6 kDa). Then, 0.5 ml (wet volume) of the adsorbent was incubated with 7.5 ml of the above mixed solution, with constant shaking for 2 h at 37°C. Finally, the supernatants were collected and analysis was performed. Experiments were conducted in triplicates.

## Results

### Characterization of adsorbents

**Figure 1:**
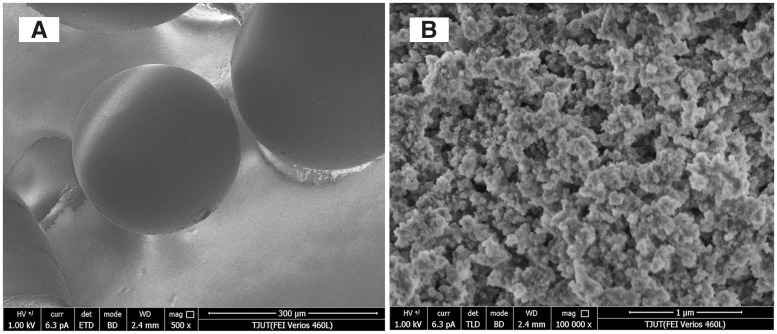
SEM micrographs of adsorbent NKU-9.

SEM images of NKU-9 are shown in Fig. 1. NKU-9 is of spherical shape in the size range from 0.2 mm to 0.8 mm, as confirmed by optical microscope. The microspheres are well formed with a highly porous structure, and clear mesopore can be seen on its surface. The pore size of the adsorbents was tested in the dry stage; therefore, the pores may be larger in practice.

**Figure 2:**
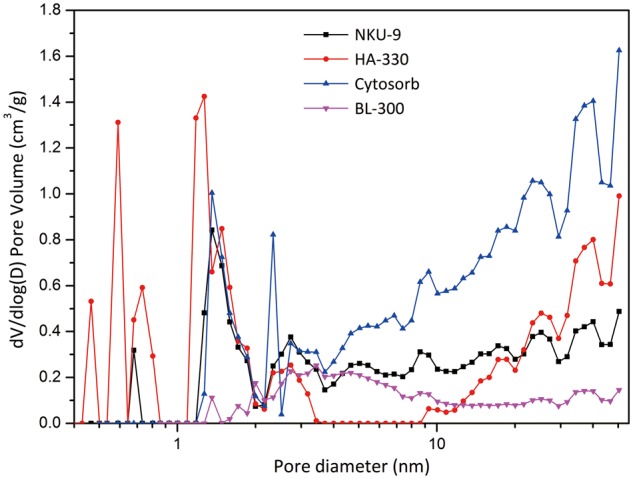
Pore size distribution of adsorbents.

Table 1 shows NKU-9 has a high specific surface area (734 m^2^/g) and relatively large average pore diameter (4.49 nm). The pore size distribution (Fig. 2) shows that NKU-9 has a wide distribution and almost equal amount of mesopores from 2 to 50 nm. In other words, NKU-9 has a homogeneous dispersed mesopores without any relative high peaks. HA-330 shows a main peak distribution in the microporous region, which results in its high specific surface area (1154 m^2^/g). In addition, HA-330 is poor in mesoporous structures, especially from a range of 3.5–9 nm. Cytosorb contains highly developed pore structure, and its mesopores mostly are larger than 10 nm. As a typical anion exchange resin, BL-300 has a concentrated distribution (a relative broad peak) between 2 and 8 nm. Therefore, NKU-9 adsorbent with a unique mesoporous structure may have some special adsorption characteristics for toxins.

**Table 1:** Physical properties of adsorbents

	Specific surface area (m^2^/g)	Pore diameter (nm)
NKU-9	734	4.49
HA-330	1154	3.34
Cytosorb	744	5.56
BL-300	174	4.96

### Adsorption of low molecular weight toxins

**Figure 3:**
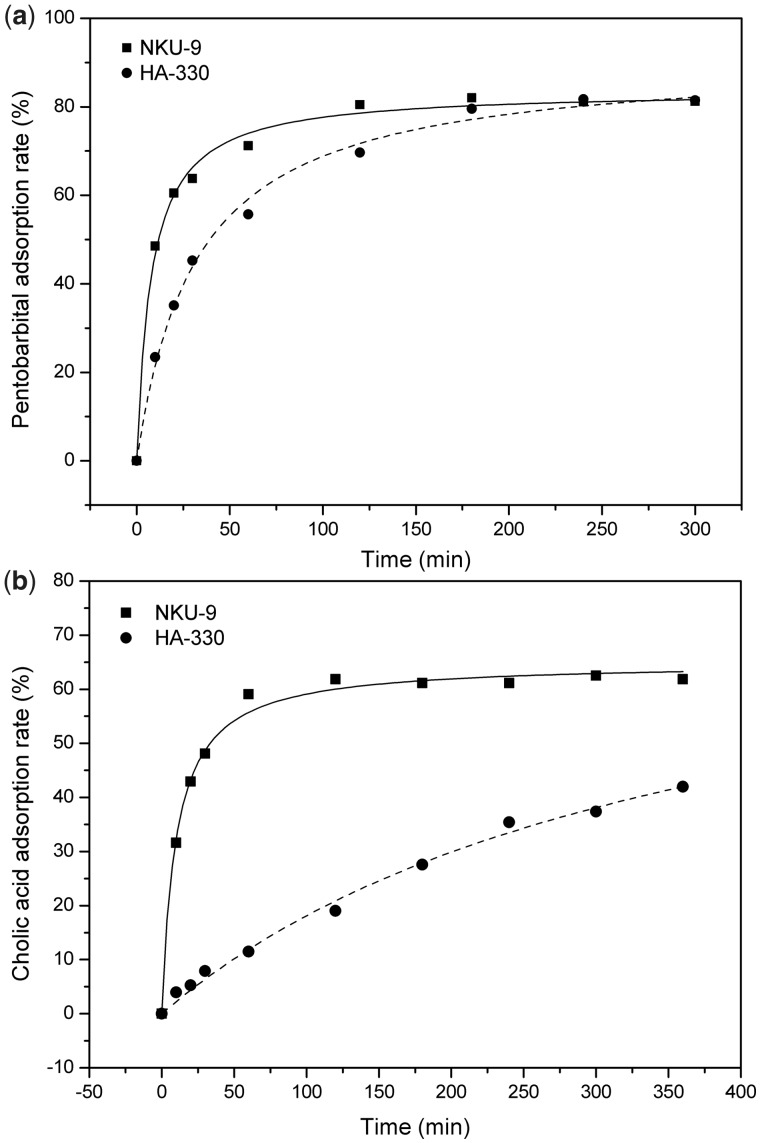
(a) Adsorption rates of pentobarbital in phosphate buffer solution. *C*_Pentobarbital_ =0.6 mg/ml; the adsorbent to solution ratio was 120 (v/v). (b) Adsorption rates of free cholic acid in phosphate buffer solution: *C*_CA _=_ _0.15 mg/ml; the adsorbent to solution ratio was 150 (v/v).

First, we investigated the adsorption properties of low molecular weight toxins of NKU-9 and HA-330 in albumin-free solution. Fig. 3 shows the time-dependent adsorption results of free pentobarbital and cholic acid in phosphate buffer solution. The curves demonstrate that NKU-9 and HA-330 both have excellent adsorption performances for pentobarbital, and their adsorption rates can all reached 80%. However, adsorbent NKU-9 reached adsorption equilibrium much faster than HA-330. In terms of free cholic acid adsorption, the adsorption performance and adsorptive velocity for low molecular weight toxins are obviously different between the two adsorbents. As shown in Fig. 3b, NKU-9 reached adsorption equilibrium much faster and had a 40% higher adsorption capacity for free bile acid than that of HA-330 in 2 h. These results suggest that adsorbent NKU-9 is more efficient and has a great advantage in removing low molecular weight toxins than HA-330.

### Adsorption of middle molecular weight toxins

**Figure 4:**
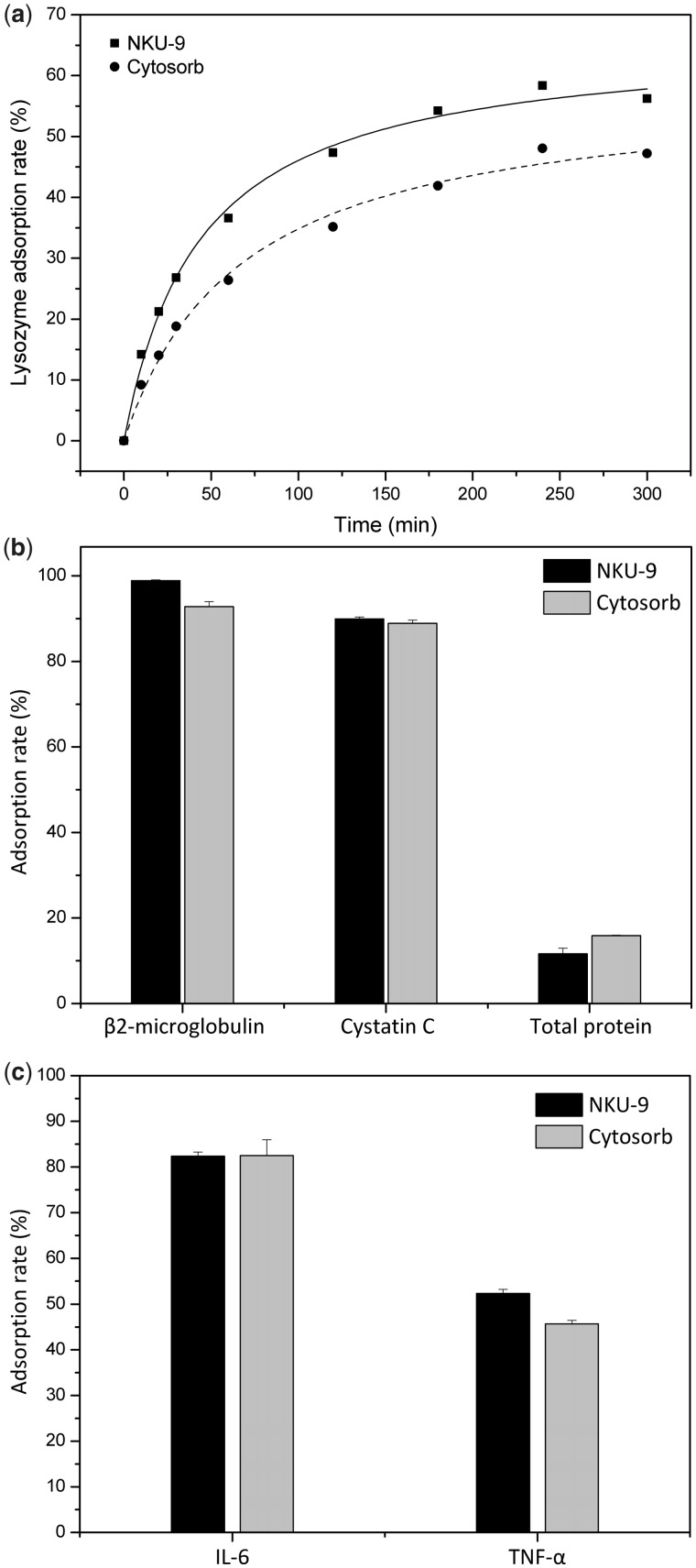
(a) Adsorption rates of lysozyme in phosphate buffer solution. *C*_lysozyme _=_ _0.6 mg/ml; the adsorbent to solution ratio was 120 (v/v). (b) Adsorption studies of plasma from dialysis patients. The adsorbent to solution ratio was 8 (v/v). (c) Adsorption studies of cytokines in spiked human plasma. The adsorbent to solution ratio was 7 (v/v).

For adsorption of middle molecular weight toxins, lysozyme (*M*_W_ = 14 kDa) was used in the study. As shown in Fig. 4, adsorption experiments suggest that NKU-9 and Cytosorb can all reach adsorption equilibrium in 4 h. NKU-9 adsorbed more lysozyme in phosphate buffer solutions than Cytosorb, and the adsorption rate of Cytosorb was about 9% lower than that of NKU-9.

The concentrations of β2-microglobulin (26.47 mg/l, *M*_W_ = 11.8 kDa) and cystatin C (6.81 mg/l, *M*_W_ = 13.3 kDa) in the plasma of dialysis patients, far exceeded the normal levels. The adsorption capacity of β2-microglobulin by NKU-9 reached 98.88% (Fig. 4b), while Cytosorb reached 92.80%. In addition, these two adsorbents both had good adsorption performances for cystatin C, and their adsorption rates were about 88%. It is worth mentioning that NKU-9 had a lower depletion of total serum proteins than Cytosorb.

In spiked human plasma, the pro-inflammatory TNF-α and IL-6 were selected for the investigation of cytokine adsorption performances. TNF-α has a molecular mass ranging from 17 kDa to 51 kDa depending on whether it is found in the monomeric, dimeric or trimeric state. As shown in the Fig. 4c, both NKU-9 and Cytosorb had well adsorption properties for TNF-α and IL-6. To be specific, the adsorption rate of adsorbent NKU-9 for IL-6 is similar to Cytosorb, while the adsorption rate of NKU-9 for TNF-α was about 6.67% higher than that of Cytosorb.

### Adsorption of albumin-bound toxins

**Figure 5:**
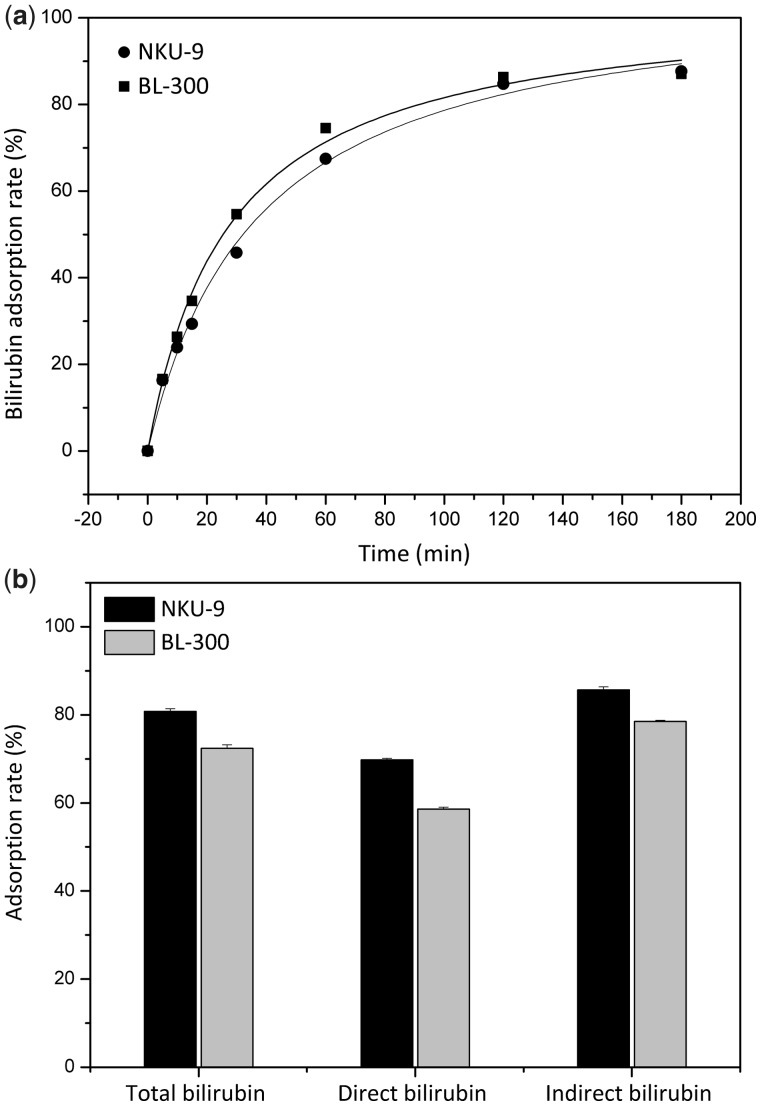
(a) Adsorption rates of bilirubin in BSA solution. *C*_BIL _=_ _150 mg/l; the adsorbent to solution ratio was 36 (v/v). (b) Adsorption studies of bilirubin in human plasma. The adsorbent to solution ratio was 5; initial plasma: *C*_TBIL _=_ _182.2 μmol/l, *C*_DBIL _=_ _56.6 μmol/l, *C*_IBIL _=_ _125.6 μmol/l.

Indirect bilirubin (albumin-bound) with sufficient albumin was added to the PBS-bilirubin solution and used for adsorption studies. The time-dependent adsorption results are shown in Fig. 5a. The curves demonstrate that, in simulated serum solution, the uptake of bilirubin by NKU-9 was quite rapid in the beginning and then slowed down with progress of time. Adsorption equilibrium was nearly reached after 120 min. The bilirubin adsorption rate reached 87%, and an adsorption capacity was 4.70 mg/ml, which correlates well with bilirubin adsorption level of BL-300 used in clinical practice.

Adsorption of albumin-bound toxins was assessed with batch experiments using human plasma with high level bilirubin of liver failure patients *in vitro*. The concentration of total bilirubin (182.2 μmol/l) in plasma of patients was much higher than the levels found in normal human, and indirect bilirubin (125.6 μmol/l) formed the majority of bilirubin toxin. Bilirubin, which is albumin-bound with an association constant of 9.5 × 10^7^M^−^^1^, was efficiently adsorbed by adsorbent NKU-9, whose total bilirubin adsorption rate and adsorption capacity reached 80.79% and 0.736 μmol/l, respectively (Fig. 5b). In comparison, BL-300, currently used to eliminate bilirubin with high efficiency in clinic, removes 72.39% total bilirubin from human plasma after treatment for 3 h.

### Adsorption in a mixture of toxin solution

When low molecular weight toxins (cholic acid), middle molecular weight toxins (IL-6) and albumin-bound toxins (bilirubin) were mixed in a simulated serum solution, adsorption capacity of the adsorbents for different molecular weight toxins indeed were different from single toxin. After incubation for 2 h, no residual cholic acid was found in all the simulated serum solutions. In other words, all the four adsorbents had perfect adsorption property for cholic acid. In addition, the adsorption rate of NKU-9 for IL-6 was 81.65%, Cytosorb was 59.75% (21.9% lower). Moreover, the adsorption rate of NKU-9 for bilirubin was 89.69%, BL-300 was79.54%, the former was 10.15% higher. The above data show that a unique adsorbent has a competitive adsorption ability in a mixture of toxins. Nevertheless, NKU-9 is the best among the four adsorbents.

## Discussion

Organ failure and sepsis often are associated with a range of different molecular weight toxic substances accumulating in the blood stream of the patient. In our study, we assume that these toxic substances can be divided into low molecular weight toxins (<1000 Da), middle molecular toxins (1000–60 000 Da) and large molecular weight toxins (>60 000 Da). In hemoperfusion, materials rich in mesopores play an important role due to the fact that target substances with middle and high molecular weight toxins can easily be removed, while microporous materials show low or no efficiency [30, 31]. Many studies showed that removal of toxic substances was effected by the relationship between the adsorbate molecular size or weight and pore size or distribution range of the adsorbent [10, 11]. In order to enhance the selectivity of an adsorbent for a particular molecular weight toxin, researchers often adjust the pore size distribution of adsorbents so that it can be concentrated in a narrow range. However, a narrow-spectrum adsorbent is often efficient only for a certain fixed molecular weight substance, but it is difficult to remove toxins with different molecular weights simultaneously with high efficiency in a one-short treatment [10]. In this study, we prepared a new family of non-ionic styrene divinylbenzene copolymers which not only possess a rich and homogeneously dispersed mesopores between 2 and 50 nm but also have a high specific surface area. We studied the adsorption performance and mechanism for different molecular weight toxins on NKU-9, and compared with other commercially available adsorbents used for clinical practice. Our results indicate that in the case of low molecular weight toxins, NKU-9 can eliminate bile acid and pentobarbital efficiently and its adsorption properties are as good as HA-330. As we know, the approximate pore size of an adsorbent for adsorption of low molecular weight toxins is 1–2 nm (micropores), so they can easily enter the inner structure of adsorbent particles freely and are bound to the matrix via hydrophilic or hydrophobic interaction. The main factor to restrict the adsorption properties of adsorbents for low molecular weight toxins is their specific surface area.HA-330, is a highly post-crosslinked resin, having an abundant micropores and a high specific surface area which can efficiently eliminate low molecular weight toxins. Our results show that HA-330 is lack of well-developed mesoporous structure; therefore, the adsorbent needs more time to reach adsorption equilibrium. In clinic, high adsorption rate and short adsorption time always imply alleviating the patients’ pain during clinical hemoperfusion. Particularly, in acute intoxication, as every minute of delay in treatment leads to an increased patient’s mortality. Adsorbent NKU-9, with rich mesopores, thus having a high adsorption rate can reach adsorption equilibrium in a relatively shorter time, at the same time, it has a high adsorption capacity for small molecules and should be a good candidate for the removal of low molecular weight toxins in clinical application.

As for adsorption of middle molecular weight toxins, NKU-9 adsorbed more lysozyme in phosphate buffer solution, which reflects its greater adsorption potential than Cytosorb which is currently used for the removal of middle molecular weight toxins in clinic. In human plasma, complex blood components have a great influence on adsorption activity by Vroman effect and would lower the adsorption rate of adsorbents for middle molecular weight toxins. The Vroman effect governs protein adsorption to the surface of a material in blood serum [32]. The small plasma proteins, which have the highest mobility, generally arrive first at the surface and are later replaced by larger ones and the less mobile proteins usually have a higher affinity for the surface of a matrix [33, 34]. Cytosorb, rich in large mesopores, allows the diffusion of more larger proteins, which may replace middle molecular weight toxins from the surface. So Cytosorb would be affected more by large proteins, and its adsorption capacity would be lower than NKU-9. Adsorption results of human plasma from dialysis patients fully confirmed this hypothesis. NKU-9 has slightly better adsorption properties for β2-microglobulin than Cytosorb. In term of cytokine, its adsorption activities are very sensitive to be affected by other plasma fraction, since there are only trace amounts existing in human plasma. Considering that Cytosorb was specially designed for cytokines adsorption, we can assume that NKU-9 is also appropriate for cytokine removal, based on its high cytokine adsorption rate which is in close proximity to Cytosorb. Furthermore, NKU-9 has less depletion of total serum proteins than Cytosorb, suggesting a better blood compatibility.

As is seen from the aspects of bilirubin adsorption, the adsorption capacity of NKU-9 is nearly as high as adsorbent BL-300 in simulated serum solution (Fig. 5*a*), and in plasma is 8.4% higher than that of anion exchange resin BL-300 (Fig. 5*b*). To our knowledge, albumin-bound toxins such as bilirubin has a hydration diameter of about 7 nm, thus a well-developed mesoporous structure may be a more decisive factor than the electrostatic interaction for its adsorption [35]. Furthermore, there are indications that strong base anion exchange resins also remove anti-coagulation agents like heparin and citrate (they are anionic), and thus lead to disturbances of the coagulation system. On the contrary, non-ionic adsorbent NKU-9 does not have these drawbacks.

**Figure 6:**
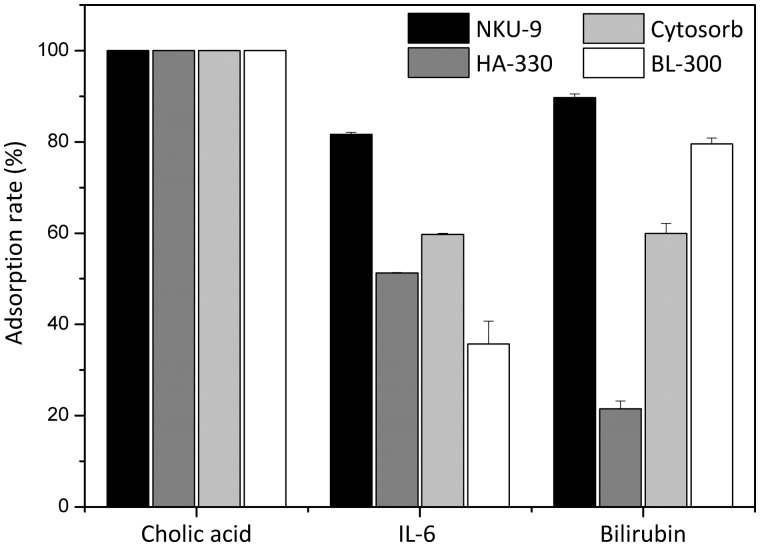
Adsorption studies of mixed solution. The adsorbent to solution ratio was 15 (v/v).

The above investigation shows that NKU-9 is an excellent adsorbent for different molecular weight toxins in each isolated solution. *In vitro* experimental results indicated that NKU-9 could highly remove cholic acid IL-6, and bilirubin in the mixture of toxins simultaneously and not be effect by the presence of other toxins which means that it is highly competitive. Furthermore, its removal capacity was even higher than Cytosorb and BL-300. It is also interesting to note that NKU-9 not only can efficiently remove various toxins in a mixture but can also bind with different molecular weights toxins (Fig. 6).

## Conclusion

In conclusion, we have successfully developed a new and unique mesoporous, bead type, non-ionic styrene–divinylbenzene copolymer adsorbent (NKU-9). The adsorbent contains a high surface area, highly developed mesoporous structure with a wide distribution and equal amount of mesopores ranging from 2 to 50 nm. The above properties are responsible for its high, fast and competitive adsorption rate for small (drugs), middle (cytokines) and high (albumin-bound) molecular weight toxins. Therefore, the present multi-functional adsorbent has a high potential application to remove various toxins safely, highly efficient and cost effective in blood purification, to treat acute diseases which conventional medical methods are difficult or have low efficiency.
